# Non-surgical Treatment of Capsular Contracture by Overfilling a Spectrum™ Saline Implant

**DOI:** 10.7759/cureus.43904

**Published:** 2023-08-22

**Authors:** Savannah Braud, Payton Yerke Hansen, Omer Sadeh, Christopher Young, Hilton Becker

**Affiliations:** 1 Surgery, Florida Atlantic University Charles E. Schmidt College of Medicine, Boca Raton, USA

**Keywords:** nonsurgical treatment, breast implant, breast, spectrum™ saline implant, capsular contracture

## Abstract

Capsular contracture is one of the most common complications following breast implant use in both aesthetic and reconstructive surgery procedures. Furthermore, capsular contracture remains one of the most common causes of reoperation in these patients. Therefore, it is important to identify and explore new treatment options to alleviate the risks associated with surgery as well as the financial burden of reoperation. We present a case of successful nonsurgical treatment of capsular contracture by overfilling a Spectrum™ adjustable saline implant followed by volume reduction. A 53-year-old patient was examined in our clinic post bilateral mastectomy and immediate reconstruction adjustable Spectrum™ saline implants in the subglandular plane. Two years postoperatively, the patient presented with Grade III capsular contracture in her right breast. Treatment was administered by overfilling the 275cc implant with 250cc in the right breast to rupture the capsule. The implant was kept overexpanded for a total of 14 days. The volume was subsequently reduced with optimal patient satisfaction. The patient has not had a reoccurrence of capsular contracture in one year. The treatment of capsular contracture by overfilling and temporarily overexpanding a Spectrum™ adjustable saline is a promising technique that warrants further investigation.

## Introduction

Breast augmentation is a surgical procedure to enhance the size and appearance of the breasts with the use of an implant. The leading complication of these procedures is capsular contracture, which has an overall incidence of 10.6%. Furthermore, it is regarded as one of the most common reasons for reoperation following breast augmentation. Capsular contracture occurs when the implant induces an immune reaction by the body resulting in fibrosis [1]. Although fibrotic tissue surrounding an implant is expected post-augmentation, capsular contracture occurs when the fibrotic tissue contracts creating an abnormally hard and painful breast. It also causes a higher nipple placement than an unaffected breast. The severity of capsular contracture is evaluated using the Baker classification system. Grade I is a soft and asymptomatic implant. Grade II is a slightly firm breast to palpation without visual changes. Grade III is a breast that is firm to palpation with visible distortions. Grade IV, the most severe, is a hard breast upon palpation with pain and distinct visual distortion. Treatment is based on grade. However, surgical treatment including capsulectomy and capsulotomy remains the mainstay treatment [2]. This approach is associated with surgical and anesthesia risks. Furthermore, there are additional monumental costs due to an additional operation. The discovery of an effective non-surgical treatment option could eliminate the risks and costs associated with reoperation; however, the prevalence of non-surgical treatments is minimal [3]. The purpose of this paper is to report a case of capsular contracture that was successfully treated in the clinic by overfilling a Spectrum™ saline Implant.

## Case presentation

A 53-year-old female with a history of recurrent right breast carcinoma opted for a bilateral mastectomy with immediate reconstruction. The breast reconstruction utilized 275 cc Spectrum^TM^ adjustable saline implants in the prepectoral plane. The implants were filled with 100cc of air intraoperatively. Air expansion of the implant facilitates better control of tissue contraction postoperatively.

The patient was evaluated on postoperative date (POD) 4 with no sign of hematoma, seroma, or infection. In the following weeks, the air was added or removed to improve the projection of the breasts while promoting tissue contraction [4]. Air injections were utilized for the first two months in-office via an injection port placed intraoperatively. All air was removed from the left breast on POD 43 and replaced with 200 cc saline to allow the weight of saline to help lower nipple placement for cosmesis. The same was done on the right breast on POD 73. The patient maintained soft and symmetrical breasts with no indication of capsular contracture.

**Figure 1:**
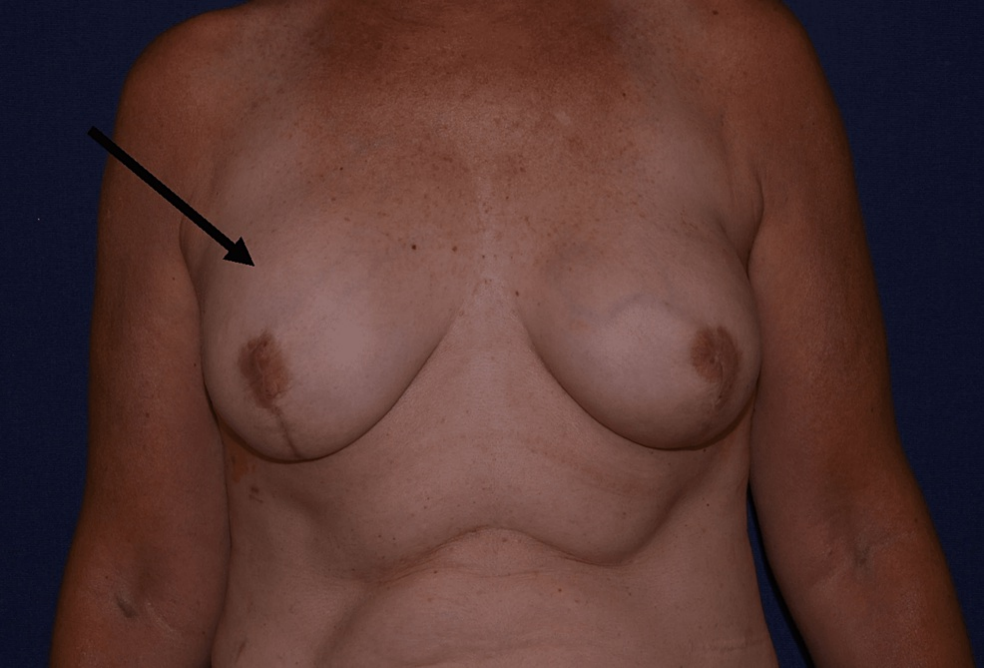
Patient presents with capsular contracture of the left breast.

**Figure 2:**
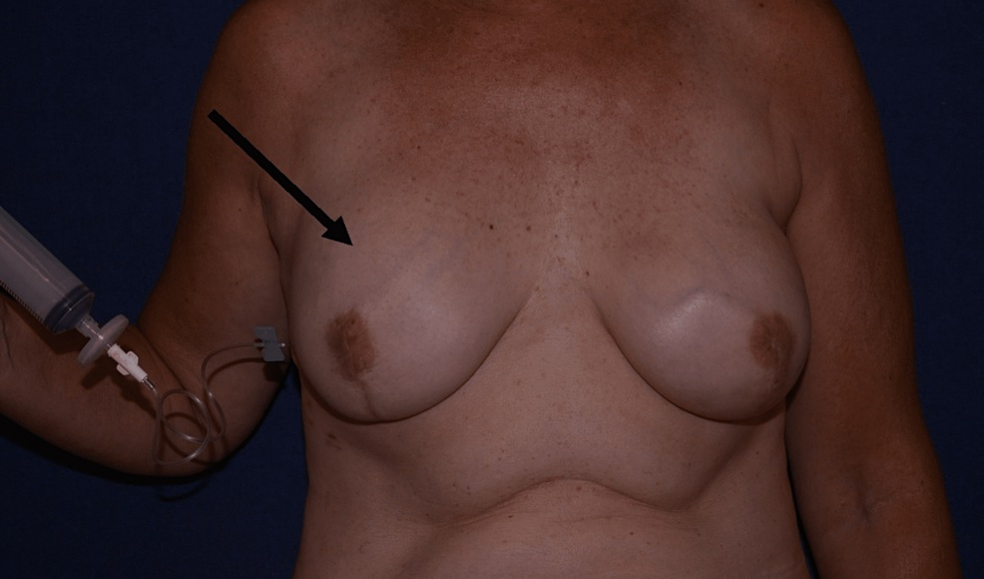
To rupture the capsule, 250cc of saline was injected via the injection port.

The patient presented to our clinic two years post-reconstruction with a Grade III capsular contracture in the right breast (Figure 1). She denied any smoking history, trauma, or infection since the initial surgery, and her current medications included vitamin supplements. A nonsurgical treatment was utilized by overexpanding the Spectrum^TM^ implant with 250cc of saline via the injection port (Figure 2). The additional saline provided enough force to rupture the capsule. After rupturing the capsule, 100cc of saline was removed to prevent patient discomfort. No analgesic or anesthetic was required during the procedure. Post-procedure pain was minimal, and the patient was advised to take ibuprofen as needed. Eleven days later, the excess saline was removed. After removing the excess fluid, her breast remained soft and nontender.

**Figure 3:**
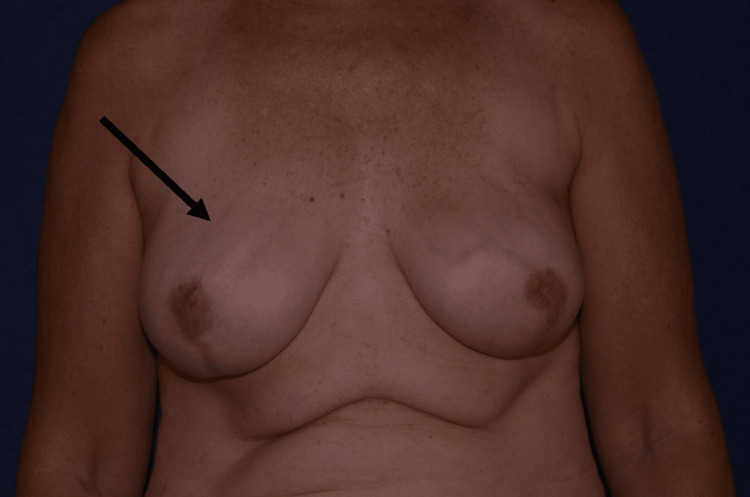
After the excess saline was removed, the patient's breast remained soft and nonpainful.

The patient has continued to follow up at the office with no further complications or recurrence of capsular contracture (Figure 3). Thus far, she has not had a recurrence of the capsular contracture a year after receiving the nonsurgical treatment.

## Discussion

The current etiology for this complication is not fully understood but is thought to be multifactorial. Studies suggest that the incision type may affect capsular contracture occurrence with a higher rate in periareolar versus transaxillary and inframammary. This may be due to alterations to the ductal system that induces an immune response from bacterial colonization [5]. In addition, smooth implants are associated with increased rates of capsular contracture compared to textured implants. With smooth implants, the lack of a textured surface leads to an improved ability for a capsule to form [6]. Subglandular implant placement is thought to have a higher incidence of capsular contracture. Without the pectoralis major, external oblique, rectus sheath, and serratus anterior muscle fascia providing a protective layer between the implant and the breast tissue, collagen deposition is more likely. Additionally, there is increased movement of the implant without the muscle holding it in place which is thought to lead to a heightened local inflammatory response [7]. It is seen more frequently after reconstructive versus cosmetic surgery, which raises the question of the impact of radiation and chemotherapy on complication development.

The traditional treatment for capsular contracture is reoperation under general anesthesia. However, capsular contracture has a high rate of recurrence. Adding an acellular dermal matrix can reduce the reoccurrence rate by roughly 20%; however, it extends operating time and increases surgical costs [8].

Furthermore, nonsurgical techniques have been reported. Compared to surgical treatment, nonsurgical methods significantly decrease overall costs by avoiding facility fees, anesthesia costs, and surgeon rates. Popular nonsurgical treatments for capsular contracture include intracapsular injection of triamcinolone, ultrasonic applications, oral Zafirlukast, Flector patch, and laser treatments [9]. While treatment of capsular contracture proved to be successful in most patients, the additional financial burden added to the original cost of the breast surgery can be challenging in addition to patient compliance with the treatment plan. Additionally, since most capsular contractures occur in reconstructive breast surgery, medication adherence can be challenging. Patients undergoing breast reconstruction secondary to breast carcinoma may be taking various oral medications in addition to chemotherapy and radiation. As a result, taking systemic medications to treat capsular contracture may be ill-advised or not possible in this patient population. This adds additional limitations for nonsurgical treatment options.

Shock wave therapy has been investigated as a nonsurgical capsular contracture treatment. This therapy has traditionally been used to remove soft connective tissue in orthopedic cases. Capsular contracture was stimulated in lab animals and treated with shock wave treatment for six consecutive weeks to identify if improvement occurred [10]. The tissue surrounding the contraction became soft with decreased collagen deposition; however, the surface of the muscle was rougher, and mean capsular scar thickness was increased. This contradicts the goal of obtaining a breast symmetrical to the unaffected breast.

In contrast, our technique involved overfilling an adjustable Spectrum^TM^ saline implant to rupture the contracted capsule. This technique may be less associated with these potential problems. Costs are minimal as the only supplies needed are syringes, butterfly needles, and saline. Patient compliance is of minimal concern since treatment is in-office. Furthermore, we were able to successfully rupture the capsule and regain breast symmetry and cosmesis with no observed recurrence of the capsular contracture. Immediately following the procedure, the patient will have breast minimal asymmetry. However, this is resolved once the excess saline is removed. Future work could include radiologic evaluation of the capsule as well as identifying the efficacy of this treatment option in a larger population size.

## Conclusions

Capsular contracture is a common complication following breast surgery and little is known about its etiology. Currently, the mainstay treatment option is surgery under general anesthesia. However, our nonsurgical method of overfilling a Spectrum™ saline implant showed to be effective in rupturing the breast capsule with no recurrence in this patient. Overall, this technique has the potential to reduce operation costs, but further investigation is warranted.
